# Dietary patterns and multiple chronic diseases in older adults

**DOI:** 10.1186/s12986-024-00814-y

**Published:** 2024-06-24

**Authors:** Danhui Mao, Gongkui Li, Moxuan Liang, Shiyun Wang, Xiaojun Ren

**Affiliations:** 1 Shanxi Bethune Hospital, Third Hospital of Shanxi Medical University, Shanxi Academy of Medical Sciences, Tongji Shanxi Hospital, Taiyuan, China; 2 Health Management and Policy Research Center, School of Management, Shanxi Medical University, Taiyuan, China

**Keywords:** Diet, Dietary pattern, Multiple chronic diseases, Latent class analysis, Older adults

## Abstract

**Background:**

The prevalence rate of multiple chronic diseases among the elderly is relatively high, posing a risk to their health and also imposing a financial burden on them. Optimal dietary patterns have positive effects on multiple chronic diseases. This study aimed to identify dietary patterns associated with multiple chronic diseases in older adults.

**Methods:**

Dietary intake was assessed through two non-consecutive 24-hour dietary recalls. The presence of multiple chronic diseases was assessed based on the existence of dyslipidemia, hypertension, chronic kidney disease, sleep disorders, diabetes, moderate or severe depressive symptoms, and cognitive impairment, with two or more of these conditions being considered. Latent class analysis was used to identify types of multiple chronic diseases, and two-step cluster analysis was used to determine individual dietary patterns. Logistic regression analysis with robust standard errors was conducted to determine the associations between dietary patterns and types of multiple chronic diseases.

**Results:**

Three dietary patterns and three types of multiple chronic diseases were identified. Individuals following a diet rich in legumes, meat, vegetables and fruits (HLMVF dietary pattern) were 59% less likely to have the cardiometabolic cognitive impairment comorbidity (CCC) than those following a diet rich in milk and eggs but with low grain intake (HME-LG) (*OR* = 0.41, 95% *CI*: 0.27–0.64, *P* < 0.001) and 66% less likely to have the especially sleep disorders comorbidity (ESC) than those following a diet rich in grains but lacking milk and eggs (HG-LME) (*OR* = 0.34, 95% *CI*: 0.14–0.87, *P* < 0.05).

**Discussion:**

The HLMVF dietary pattern may serve as a healthy dietary pattern to reduce the incidence of multiple chronic diseases and should be promoted among the older adult population.

## Introduction

Patients with multiple chronic diseases suffer from reduced life expectancy because of the long course and complex etiology of chronic diseases. Multiple chronic diseases pose a greater risk of disability and death than a single chronic disease among older adults, affecting their physical and mental health and imposing a financial burden on them [1]. Therefore, the prevention and control of multiple chronic diseases is one of the important goals of primary health services.

Multiple chronic diseases have the characteristics of complex underlying mechanisms, a high degree of correlation with each other, and multiple levels of influencing factors. In general, the prevalence of multiple chronic diseases is closely related to demographic, lifestyle, psychological, and societal characteristics. The most relevant demographic characteristics are age, gender, education level, and economic level of the living area. The prevalence of multiple chronic diseases among older adults is relatively high. Globally, 82% of the multiple chronic patients are aged 85 years or older, 65% of them are aged 65–84 years, and 30% of them are aged 45–64 years. Furthermore, there are more women patients than men; most patients have lower education levels and are living in economically disadvantaged areas [2]. In addition, similar to a single chronic disease, the occurrence and development of multiple chronic diseases are closely related to lifestyles, especially dietary patterns and physical activities [3].

As changeable factors in the prevention and control of multiple chronic diseases, food and nutrients work in coordination and in a dynamic system with complex interaction. The dietary patterns can reflect the nutrition status of the body to a certain extent. Generally, healthy dietary patterns can improve many chronic symptoms such as improving kidney function and reducing serum uric acid levels simultaneously [4, 5]. For example, obesity is one of the most important chronic metabolic diseases that is associated with many comorbidities. The Mediterranean, dietary approach to stop hypertension (DASH), Atkins, Ernest, and regional dietary patterns have the effect of decreasing obesity [6–10]. In addition, the Mediterranean and DASH dietary patterns are internationally recognized dietary patterns that can be used for the prevention and control of multiple chronic diseases [11]. Currently, the research on the relationship between dietary patterns and chronic diseases is mainly focused on the relationship between dietary patterns and a single chronic disease, dietary patterns and multiple chronic symptoms, and the Mediterranean or DASH dietary patterns and multiple chronic diseases. However, there is a lack of exploration of the relationship between other dietary patterns and multiple chronic diseases. Therefore, this study aimed to evaluate whether other dietary patterns were associated with multiple chronic diseases.

## Methods

### Study design and population

In order to cover a wide range of chronic disease types as much as possible, we chose subjects from the National Health and Nutrition Examination Survey (NHANES) from 2011 to 2014. The detailed description of the study protocol is available at https://www.cdc.gov/nchs/nhanes/. We excluded those with incomplete data (including sociodemographic characteristics, diet, lifestyle characteristics and health characteristics), those aged below 60 years, and those who have one kind of chronic disease or more. As a result, 2165 subjects were included in the study (see Table 1 for detail).

**Table 1:** Characteristics of the subjects

	*N*(%) or Mean (Standard Error)
Mean age	69.30 (6.71)
Females	1113 (51.4)
Marital status	
Married Widowed Divorced Separated Never married Living with partner	1236 (57.1) 383 (17.7) 316 (14.6) 57 (2.6) 119 (5.5) 54 (2.5)
Education level	
Less than 9th grade 9-11th grade High school graduate/GED or equivalent Some college or AA degree College graduate or above	195 (9.0) 290 (13.4) 516 (23.8) 639 (29.5) 525 (24.2)
Mean family PIR	2.68 (1.60)
Drank less than 12 glasses of wine per year	650 (30.0)
Didn’t use tobacco/nicotine in last 5 days	296 (13.7)
Reached the WHO physical activity level	1085 (50.1)
BMI	26.86 (9.06)
Had dyslipidemia	1455 (67.2)
Had hypertension	1732 (80.0)
Had chronic kidney disease	393 (18.2)
Had sleep disorders	251 (11.6)
Had diabetes mellitus	680 (31.4)
Had moderate or severe depressive symptoms	194 (9.0)
Had low cognitive functions	543 (25.1)

The survey obtained the approval of the ethics committee of the National Center for Health Statistics in USA. All subjects signed informed consent prior to the investigation.

### Dietary food consumption assessment

Then, 9 types of foods (g) consumed within two days were selected, which are commonly included in the dietary patterns of American adults (see Table 2 for detail).

**Table 2:** Dietary food intakes in the classes of dietary patterns

Intake (g per two day)	Pattern I HLMVF	Pattern II HG-LME	Pattern III HME-LG	F	*P*
Milk and milk products	336.00 (226.12)	96.58 (22.20)	753.40 (643.44)	422.29	< 0.001
Meats and meats products	232.1 (199.67)	191.42 (151.08)	194.86 (141.10)	5.41	0.005
Eggs and eggs products	73.41 (78.16)	26.73 (40.64)	111.62 (107.32)	293.16	< 0.001
Legumes and legumes products	151.11 (131.31)	20.59 (35.00)	21.48 (31.45)	559.91	< 0.001
Grains and grains products	250.20 (191.74)	304.09 (213.88)	157.35 (117.09)	167.23	< 0.001
Fruits and fruits products	382.67 (314.68)	175.82 (176.75)	217.98 (146.88)	99.19	< 0.001
Vegetables and vegetables products	399.12 (349.21)	180.68 (145.74)	167.28 (125.97)	149.42	< 0.001
Fats and fats products	143.51 (327.69)	12.32 (17.15)	95.51 (85.34)	206.30	< 0.001
Sweet products and sugar beverages	1304.34 (774.83)	1785.05 (930.66)	951.76 (547.78)	281.53	< 0.001

### Multiple chronic diseases assessment

In this study, dyslipidemia, hypertension, chronic kidney disease, sleep disorders, diabetes mellitus, moderate or severe depressive symptoms and low cognitive functions were assessed. The presence of multiple chronic diseases were assessed according to the WHO definition, which considers the coexistence of two or more chronic conditions in the same individual. The dyslipidemia of the subjects were assessed as at least one of the following: (1) high-density lipoprotein cholesterol < 40 mg/dL (1.03mmol/L) in men or < 50 mg/ dL (1.29mmol/L) in women [12], (2) the subject has ever been told by a doctor or other health professional that his or her blood cholesterol level was high. The hypertension of the subjects was assessed as at least one of the following: (1) the average systolic blood pressure of three measurements was over 130 or the average diastolic blood pressure of there measurements was over 80, (2) the subject has ever been told by a doctor or other health professional that he or she has hypertension. The chronic kidney disease of the subjects were assessed by an albumin-to-creatinine ratio of ≥ 30. The sleep disorders of the subjects were assessed based on whether the subject had ever been told by a doctor or other health professional that he or she had sleep disorders. The diabetes mellitus of the subjects was assessed as at least one of the following: (1) the fasting blood glucose was ≥ 126 mg/dL, (2) the two-hour oral glucose tolerance test was ≥ 200 mg/dL, (3) the subject has ever been told by a doctor or other health professional that he or she has diabetes mellitus. The depressive symptoms of the subjects were assessed by using the Patient Health Questionnaire-9 (PHQ-9). The PHQ-9 score range was from 0 to 27. When the PHQ-9 score was ranging 10 to 27, it means the individual had a moderate or severe depressive symptoms. The higher the PHQ-9 score, the more severe the symptoms of depression. Details about the PHQ-9 are available elsewhere [13]. The cognitive functions of the subjects were assessed by the Consortium to Establish a Registry for Alzheimer’s disease test, the Animal Fluency test and the Digit Symbol Substitution test. The subjects with low cognitive functions were assessed by the overall cognitive test score after normalization which was below minimum quartile (*P*_*25*_) based on Kroenke et al. (2001) [14].

### Covariates

The other variables in the study were sociodemographic characteristics, lifestyle characteristics and other health characteristics. Sociodemographic characteristics included age, gender, marital status, education level, family poverty-to-income ratio (PIR), which is the ratio of family income to the poverty threshold. Lifestyle characteristics included alcohol use status, smoking status, and physical activity level. Other Health characteristics included Body Mass Index (BMI). We classified alcohol use status into two types: those who drank at least 12 glasses of wine per year and those who drank less than 12 glasses of wine per year. We classified smoking status into two types as had used tobacco/nicotine last 5 days and did not use tobacco/nicotine in the last 5 days. The physical activity level of the subjects were assessed as reached the WHO recommendation to set the cut-off values for moderate-intensity physical activity by 150 min per week, vigorous-intensity physical activities by 75 min per week, and a total of 600 METs per week, according to the Global Physical Activity Questionnaire guideline [15]. See Table 1 for detail.

### Statistical analysis

Descriptive statistics were conducted to estimate the Mean (Standard Deviation) for continuous variables such as age, PIR, and BMI. Frequency (Proportion) was calculated for categorical variables such as gender, marital status, education level, alcohol use status, smoking status, physical activity level, dyslipidemia, hypertension, chronic kidney disease, sleep disorders, diabetes mellitus, moderate or severe depressive symptoms, and low cognitive functions status. To determine differences in the distribution of chronic disease status with respect to demographic characteristics, *T* tests, *F* tests and *χ*^*2*^ tests were utilized, followed by post hoc multiple comparisons using the Bonferroni test. Pearson or Spearman correlation was employed to analyze variable correlations. Latent class analysis (LCA) was used to identify subgroups of individuals with multiple chronic diseases. Two-step cluster (TSC) was used for identifying individuals’ dietary patterns. The association between dietary patterns and subgroups of multiple chronic diseases was determined using logistic regression with robust standard errors. All tests were based on two-sided tests with a 95% confidence interval.

## Results

### Demographic characteristics

The subjects’ characteristics are presented in Table 1. Approximately half of the subjects were female (51.4%), 57.1% of the subjects were married, 53.7% of the subjects had some college or AA degree and above, 30.0% of the subjects drank less than 12 glasses of wine per year, 13.7% of the subjects had not use tobacco/nicotine in last 5 days, and 50.1% met the WHO physical activity recommendations. The subjects were an average of 69.30 years of age (SE = 6.71), anaverage family poverty-to-income ratio (PIR) of 2.68 (SE = 1.60), and an average body mass index (BMI) of 26.86 (SE = 9.06). More than half of the subjects had dyslipidemia (67.2%), 80.0% of the subjects had hypertension, 18.2% of the subjects had chronic kidney disease, 11.6% of the subjects had sleep disorders, 31.4% of the subjects had diabetes mellitus, 9.0% of the subjects had moderate or severe depressive symptoms, 25.1% of the subjects had low cognitive functions.

### The clusters of multiple chronic diseases

The latent classes of the subjects’ multiple chronic diseases were examined. The LCA model fitting parameters are listed in Table 3. The model fit information for the five different models was listed, ranging from Model 1 to Model 5, is provided. In terms of the Lo-Mendell-Rubin likelihood ratio (LMRT) and Bootstrap likelihood ratio (BLRT), the *P*-values for the Model 2, 3 and 4 model were both less than 0.05 (statistically significant). There were higher Akaike Information Criterion (AIC) and Bayesian Information Criterion (BIC) in Model 2 when it compared with Model 3 and 4. The entropy in Model 3 was 0.610 which was higher than in Model 4. It was shown that the accuracy of classification was higher than 80.00% [16]. Model 3 model was better than the other models in this study.

**Table 3:** Fit indices for the Model1 through 5 models

Model	Log L^^^	AIC^^^	BIC^^^	aBIC^^^	entropy	BLRT^^^	LMRT^^^	Class probabilities
1	-7476.281	14966.561	15006.323	14984.083				
2	-7332.515	14695.029	14780.232	14732.575	0.452	<0.001	<0.001	0.326/0.674
3	-7310.950	14667.900	14798.544	14725.470	0.610	<0.001	<0.001	0.334/0.0676/0.599
4	-7291.383	14644.767	14820.852	14722.361	0.505	0.020	0.040	0.061/0.183/0.577/0.178
5	-7279.964	14637.929	14859.456	14735.548	0.593	0.060	0.204	0.041/0.081/0.187/0.158/0.533

^^^ LogL, Log likelihood. AIC, Akaike information criterion. BIC, Bayesian information criterion. aBIC, sample size-adjusted Bayesian information criterion. BLRT, Bootstrap likelihood ratio test. LMRT, Lo-Mendell-Rubin likelihood ratio test. LogL stands for log likelihood. AIC is a metric used to compare the relative quality of statistical models, balancing model fit and complexity to select an effective model that explains data without overfitting. BIC selects models from a finite set by penalizing complexity and emphasizing model fit. BLRT compares nested models in statistical modeling. LMRT compares different parameterizations in mixture models, aiding researchers in selecting the best model for their data

**Fig. 1:**
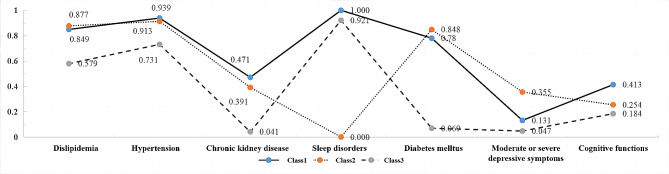
Class proportions and class-specific probabilities from three-latent-class model of multiple chronic diseases

Class proportions and class-specific probabilities from three-latent-class model of multiple chronic diseases are presented in Fig. 1. Among the subjects, 14.4% (*n* = 312) belonged to Class 1, 48.8% (*n* = 1056) belonged to Class 2, 36.8% (*n* = 796) belonged to Class 3. Class 1 was characterized by subjects with low probabilities of having moderate or severe depressive symptoms and high probabilities of having low cognitive functions (*P* < 0.05). Class 2 was characterized by subjects with low probabilities of having sleep disorders and moderate probabilities of experiencing moderate or severe depressive symptoms (*P* < 0.05). Class 3 was characterized by subjects with low probabilities of having dyslipidemia, hypertension, chronic kidney disease, diabetes mellitus, and moderate or severe depressive symptoms (*P* < 0.05). Class 3 had a higher probabilities of having sleep disorders than Class 2 (*P* < 0.05). Therefore Class 1 was labeled as cardiometabolic cognitive impairment comorbidity (CCC), Class 2 was labeled cardiometabolic depression comorbidity (CDC), and Class 3 was labeled especially sleep disorders comorbidity (ESC). Subjects with CCC were somewhat older than others, and with CDC were somewhat older than subjects with ESC (*F* = 22.12, *P* < 0.05). Subjects with ESC had somewhat lower family PIR scores than others (*F* = 35.70, *P* < 0.05). Additionally, there was an association between gender, marital status, education level, alcohol use status, physical activity level, and the class of multiple chronic diseases, with *χ*^*2*^ values respectively of 8.94, 31.10, 73.65, 12.52, 24.67 (*P* < 0.05). There was no association between BMI and smoking status (*P* > 0.05).

### The clusters of dietary patterns

The clusters of the subjects’ dietary patterns was examined. The TSC model fitting parameters are listed in Table 4. The model fit information for the ten different models was listed, ranging from Model 1 to Model 10. In terms of the Target distance measurement ratio (TDMR), the values were relatively higher in Model 2 and Model 3. The Bayesian Information Criterion (BIC) for the Model 3 was lower than for Model 2.

**Table 4:** Fit indices for the class 1 through 10 Models

Model	BCI^@^	ΔBCI^@^	ΔBCI ratio^@^	TDMR^@^
1	13639.72			
2	11124.99	-2514.73	1.00	2.42
3	10164.66	-960.33	0.38	2.21
4	9804.98	-359.68	0.14	1.23
5	9537.63	-267.36	0.11	1.02
6	9279.34	-258.29	0.10	1.08
7	9051.41	-227.93	0.09	1.26
8	8900.03	-151.38	0.06	1.28
9	8811.55	-88.48	0.04	1.08
10	8738.91	-72.64	0.03	1.02

^@^ BIC, Bayesian information criterion. ΔBCI, the change of Bayesian information criterion. ΔBCI ratio, the change ratio of Bayesian information criterion. TDMR, Target distance measurement ratio

The class proportions in Model 3 were better than the other models in this study. Among the subjects, 7.9% (*n* = 172) had the Pattern I dietary pattern, 50.8% (*n* = 1099) had the had the Pattern II dietary pattern, and 41.3% (*n* = 893) had the Pattern III dietary pattern. The intakes of foods for each identified class of dietary pattern was showed in Table 2. Pattern I was characterized by subjects with middle intake of milk and milk products, eggs and eggs products, grains and grains products, sweet products and sugar beverages (sugars, sweet, and beverages), and highest intake of legumes and legumes products (dry beans peas other legumes nuts and seeds), meats and meats products (meat, poultry, fish and mixtures), fruits and fruits products, vegetables and vegetables products, fats and fats products (fats, oils and salad dressings). Pattern II was characterized by subjects with lowest intake of milk and milk products, eggs and eggs products, fats and fats products, fruits and fruits products, and lower intake of meats and meats products, legumes and legumes products, vegetables and vegetables products, and highest intake of grains and grains products, sweet products and sugar beverages. Pattern III was characterized by subjects with highest intake of milk and milk products, eggs and eggs products, lower intake of meats and meats products, legumes and legumes products, vegetables and vegetables products, middle intake of fruits and fruits products, fats and fats products, lowest intake of grains and grains, sweet products and sugar beverages. Therefore Pattern I was labeled Highest in legumes and legumes products, meats and meats products, fruits and fruits products, vegetables and vegetables products, and fats and fats products (HLMVF). Pattern II was labeled Highest grains, sweet products, and Lowest milk and milk products, eggs and eggs products, fruits and fruits products, fats and fats products (HG-LME). Pattern III was labeled Highest milk and milk products, eggs and eggs products, and Lowest grains and grains products, sweet products and sugar beverages (HME-LG). There was an association between the class of dietary patterns and the class of multiple chronic diseases with *χ*^*2*^ = 20.82 (*P* < 0.001).

### The relationship between dietary patterns and multiple chronic diseases

Since the ESC group had relatively low levels of almost all diseases, it was used as the reference group for comparison. As observed in Table 5, older adults adhering to a HLMVF dietary pattern were 59% less likely (*OR* = 0.41, 95% *CI*: 0.27–0.64, *P* ≤ 0.001) to being CCC and 66% less likely (*OR* = 0.34, 95% *CI*: 0.14–0.87, *P*<0.05) to being CDC compared to those following a HME-LG dietary pattern. In the primary model, which only controlled for demographic variables, the *OR* values for CCC (*OR* = 0.41–0.44) and CDC (*OR* = 0.28–0.34) were both statistically significant (*P* <0.05). This significance remained consistent when additional physical characteristics or lifestyle factors were taken into account in the model (see details in Table 5).

**Table 5:** Association between dietary patterns and multiple chronic diseases

	Odds Ratio (95% Confidence Interval)^∼^			
	HLMVF dietary pattern^!%^		HG-LME dietary pattern^!%^	
	CCC^!^	CDC^!^	CCC^!^	CDC^!^
Unadjusted Model	0.41(0.27,0.64)^* * *^	0.34(0.14,0.87)^*^	0.89(0.73,1.09)	0.99(0.69,1.41)
Primary Model^#^	0.44(0.28,0.69)^* * *^	0.30(0.12,0.77)^*^	0.94(0.76,1.15)	1.01(0.70,1.45)
Sensitivity Analyses				
+ control for physical characteristics ^#,$^	0.44(0.28,0.70)^* * *^	0.28(0.11,0.72)^* *^	0.95(0.77,1.17)	0.91(0.62,1.32)
+ control for other lifestyle characteristics^#, &^	0.44(0.28,0.70)^* * *^	0.30(0.12,0.78)^*^	0.93(0.76,1.14)	0.99(0.69,1.42)

^!^CCC, Low depressive symptoms High others Especially low cognitive functions. CDC, Low sleep disorders High others. ESC, High sleep disorders low other disease. HLMVF, Highest legumes and legumes products, meats and meats products, fruits and fruits products, vegetables and vegetables products, and fats and fats products. HG-LME, Highest grains, sweet products, and Lowest milk and milk products, eggs and eggs products, fruits and fruits products, fats and fats products. HME-LG, Highest milk and milk products, eggs and eggs products, and Lowest grains and grains products, sweet products and sugar beverages.

^∼^ The ESC was the reference.

^%^ The HME-LG was the reference.

^#^ Estimated using Logistic regression. Model control for gender, age, educational level, marital status, family PIR.

^$^ Models additionally control for BMI.

^&^ Models additionally control for smoking status and physical activity level.

^*^P<0.05, ^**^P<0.01, ^***^P<0.001

## Discussion

The purpose of this study was to identify dietary patterns associated with multiple chronic diseases in the older adult population. Through Latent Class Analysis, the study identified three different types of multiple chronic diseases and three different types of dietary patterns. Among these three dietary patterns, the HLMVF dietary pattern was found to be beneficial in reducing the incidence of CCC and CDC types of multiple chronic diseases. The HLMVF dietary pattern is characterized by high levels of nutrient intake, particularly protein from both plant-based sources such as legumes and legume products, and animal-based sources like meat and meat products. It also features vitamin and mineral-rich foods, such as fruits and fruit products, as well as vegetables and vegetable products. The common characteristics of CCC and CDC type multiple chronic diseases are increased occurence of cognitive impairment, dyslipidemia, hypertension, chronic kidney disease, and diabetes.

According to current research, the understanding of relationship between different types of dietary patterns and various types of multiple chronic diseases is still relatively limited in terms of study experiences. Therefore, researchers can currently only compare the findings with studies that focus on individual chronic diseases. The results of this study suggest that simultaneously consuming a dietary pattern rich in high-quality protein, vitamins, and minerals may lower the incidence of several chronic diseases, including cognitive impairment, dyslipidemia, hypertension, chronic kidney disease, and diabetes.

The findings of this study align with previous research conducted in related areas. For instance, a study by Xu et al. (2022) discovered a negative correlation between increased protein intake and cognitive impairment, emphasizing the potential benefits of adequate protein consumption for cognitive function [17]. Similarly, Li et al. (2020) found a positive association between dietary protein intake and cognitive function among adults aged 60 and above [18]. These findings suggest that protein intake may play a significant role in preserving cognitive health. Furthermore, earlier research has demonstrated associations between various vitamins and minerals and the occurrence of specific chronic diseases [19–23]. These nutrients possess antioxidant properties, aiding in neutralization of harmful free radicals and reduction of oxidative stress within the body. Additionally, some of these vitamins and minerals exhibit anti-inflammatory effects, regulating the inflammatory response and potentially mitigating the impact of chronic inflammation on various chronic diseases.

The intake of protein from various sources has been linked to the potential improvement of multiple chronic diseases, such as CCC and CDC, which can be further explained as follows: Firstly, research suggests that increasing protein intake is associated with a reduction of sarcopenia, the progressive loss of muscle mass and strength commonly observed in older adults, and is a significant component of frailty and disability [24–26]. This is particularly important for patients with multiple chronic diseases as they often experience muscle wasting due to inactivity or other factors. Adequate protein intake can help preserve muscle mass, reduce falls and fractures, and enhance overall physical performance [27]. Moreover, protein plays a crucial role in the repair and regeneration of tissues such as skin, bone, and cartilage, which are frequently affected by chronic diseases. Secondly, protein is essential for maintaining immune function, especially in older adults, whose immune systems may become compromised due to chronic inflammation or immunosenescence [28]. The study has shown that a diet including a variety of high-quality protein sources can enhance immune function and reduce the likelihood of infections [29] and other complications associated with chronic conditions. Thirdly, consuming protein from diverse sources can promote the richness and diversity of gut microbiota, which is increasingly recognized as a crucial factor in maintaining health and preventing chronic diseases. Gut microbiota play significant roles in nutrient digestion, absorption, metabolism, immune regulation, and inflammation [30]. Studies have indicated that a diet rich in plant-based protein sources like legumes and nuts can increase the abundance of beneficial gut bacteria, such as *Bifidobacterium* and *Lactobacillus*, while reducing the levels of harmful bacteria like *Clostridium* and *Enterobacteriaceae* [31]. This can have positive effects on overall health and reduce chronic diseases, including multiple chronic diseases such as CCC and CDC. In conclusion, consuming protein from a variety of sources can benefit patients with multiple chronic diseases such as CCC and CDC by preserving muscle mass and function, enhancing immune function, and promoting the richness and diversity of gut microbiota.

The potential reasons for the improvement of multiple chronic diseases, such as CCC and CDC, through the intake of rich vitamins and minerals can be summarized as follows: Firstly, many vitamins and minerals possess antioxidant properties. They help neutralize harmful free radicals in the body, reduce oxidative stress, and protect cells from damage caused by reactive oxygen species. Oxidative stress is believed to play a role in the development and progression of various chronic diseases, including cardiovascular diseases and neurodegenerative disorders [32]. Adequate intake of antioxidants can help mitigate oxidative stress and potentially improve the outcomes of these diseases. Secondly, some vitamins and minerals exhibit anti-inflammatory properties. Chronic inflammation is a common underlying factor in many chronic diseases, including cardiovascular diseases, metabolic disorders, and autoimmune conditions. By regulating the production of pro-inflammatory molecules and promoting the synthesis of anti-inflammatory compounds, these nutrients can help alleviate chronic inflammation and reduce the incidence or severity of associated diseases [33]. Thirdly, certain vitamins and minerals are involved in blood glucose metabolism and insulin secretion regulation. They play essential roles in carbohydrate metabolism, assisting in the breakdown, absorption, and utilization of glucose from the diet. Adequate levels of these nutrients contribute to maintaining stable blood sugar levels, improving insulin sensitivity, and reducing the incidence of developing type 2 diabetes [34]. Additionally, well-controlled blood glucose levels are crucial for managing other chronic diseases, such as cardiovascular diseases and kidney disorders [35]. Finally, vitamins and minerals also play an important role in promoting the richness and diversity of the gut microbiota. Emerging research has highlighted the importance of a healthy gut microbiome in maintaining overall health and preventing chronic diseases. Certain vitamins serve as essential cofactors for the growth and metabolism of beneficial gut bacteria [36]. Meanwhile, minerals like magnesium can act as prebiotics, providing nourishment for beneficial microbial species [37]. A diverse and balanced gut microbiota composition has been associated with improved immune function, reduced inflammation, and better overall metabolic health. In summary, the intake of rich vitamins and minerals can provide antioxidant and anti-inflammatory effects, contribute to blood glucose regulation, and promote a healthy gut microbiota composition, all of which can contribute to the improvement of multiple chronic diseases, including CCC and CDC.

Therefore, the HLMVF dietary pattern may play a role in maintaining muscle protein, improving immune function, reducing inflammation, and enhancing overall metabolism, thereby decreasing the risk of multiple chronic diseases among the older adults.

### Strengths and limitations

This study has some limitations. Firstly, the sample population is from the United States, and therefore, the dietary patterns identified may not be directly applicable to other cultures. Secondly, as a cross-sectional study, it cannot establish a causal relationship between dietary patterns and multiple chronic diseases. Finally, due to data limitations, this study did not consider other chronic diseases. Hence, further large-scale cohort studies are required to validate the findingd in a worldwide population.

## Conclusion

In conclusion, the HLMVF dietary pattern may be a healthy dietary pattern for reducing multiple chronic diseases in older adults. The HLMVF dietary pattern is characterized by a variety of high-quality protein, lipid, vitamin, and mineral-rich foods. Future research should adapt the HLMVF dietary pattern to local cultures and investigate its causal relationship with multiple chronic diseases in the worldwide population.

## Data Availability

Our data availability at https://www.cdc.gov/nchs/nhanes/.
